# A Global, Multi-Disciplinary, Multi-Sectorial Initiative to Combat Leptospirosis: Global Leptospirosis Environmental Action Network (GLEAN)

**DOI:** 10.3390/ijerph110606000

**Published:** 2014-06-05

**Authors:** Kara N. Durski, Michel Jancloes, Tej Chowdhary, Eric Bertherat

**Affiliations:** 1 World Health Organization, Avenue Appia 20, 1211 Geneva 27, Switzerland; E-Mail: bertherate@who.int; 2 Health and Climate Foundation, 1425 K St. NW Suite 350, Washington, DC 20005, USA; E-Mail: michel.jancloes@gmail.com; 3 UNANGO, Route de Ferney 150, 1211 Genève, Switzerland; E-Mail: chowdharyt@unango.org

**Keywords:** leptospirosis, One Health, outbreak

## Abstract

Leptospirosis has emerged as a major public health problem in both animals and humans. The true burden of this epidemic and endemic disease is likely to be grossly under-estimated due to the non-specific clinical presentations of the disease and the difficulty of laboratory confirmation. The complexity that surrounds the transmission dynamics, particularly in epidemic situations, requires a coordinated, multi-disciplinary effort. Therefore, the Global Leptospirosis Environmental Action Network (GLEAN) was developed to improve global and local strategies of how to predict, prevent, detect, and intervene in leptospirosis outbreaks in order to prevent and control leptospirosis in high-risk populations.

## 1. Leptospirosis, an Emerging Public Health Problem

Leptospirosis has emerged as a major public health problem for both animals and humans in developed and developing countries alike with the largest burden occurring in temperate and tropical countries. It is a bacterial disease with multiple, complex modes of transmission, numerous hosts, a multitude of pathogenic serovars, various clinical manifestations, non-specific symptoms, and difficulty in early diagnosis. It is present in domestic animals, small mammals and the natural environment; and if left untreated, it has a high potential for human mortality. Furthermore, the sensitivity to certain environmental conditions suggests that climate change (in particular floods and hurricanes) and population drift from rural to peri-urban and urban areas all impact the nature of the disease and may increase the magnitude and severity of outbreaks. To date there is a lack of clarity surrounding the disease, its global burden, effective case management (including diagnosis and treatment), the dynamic relationship between animals, humans, and the environment, methods for effective outbreak prevention, detection and response, and its economic impact [1,2,3,4,5,6,7].

Over the past two decades multiple leptospirosis groups have been created and initiatives undertaken to improve our understanding and awareness of the disease. In 1994, the International Leptospirosis Society (ILS) was created to promote knowledge of leptospirosis through the organization of regional and global leptospirosis meetings. The focus of the ILS is on advancing the scientific knowledge of the disease. In December 2009, the World Health Organization (WHO) launched the Leptospirosis Burden Epidemiology Reference Group (LERG) with the goal of estimating the global burden of Leptospirosis and increasing awareness of and commitment to the disease in developing countries [8]. Despite the public health importance of leptospirosis, until now, there has not been a global, one-health approach for the prevention and control of the disease. The purpose of this paper is to present an innovative, global network that has been developed by experts and institutions from many different disciplines and sectors. 

## 2. The Global Leptospirosis Environmental Action Network (Glean)

GLEAN was launched in 2010. It gathered representatives from international organizations and foundations as well as researchers to form a unique multi-disciplinary, multi-sectorial collaboration which builds upon the leptospirosis efforts that have already taken place. Its objective is to develop innovative and practical solutions to limit the impact of leptospirosis outbreaks. The initiative provides direction and coordination to fill the many gaps in leptospirosis knowledge in association with the ILS, with the ultimate goal of translating the research findings into operational guidance for communities and countries affected by leptospirosis outbreaks. GLEAN offers an opportunity to strengthen current public health strategies and mitigate the impact of leptospirosis outbreaks in populations at high risk. It also creates a forum to develop new advocacy and funding opportunities for leptospirosis, and offers further support for capacity building, training and technology transfer, as needed.

The GLEAN initiative was modeled on the Meningitis Environmental Risk Information Technologies (MERIT) Initiative. MERIT is a successful initiative spearheaded by WHO and the Health and Climate Foundation (HCF) to bring together international organizations, research institutes, and members of the environmental, public health and epidemiological communities with the goal of reducing the burden of epidemic meningococcal meningitis across Africa’s meningitis belt. The GLEAN initiative builds upon the strengths of the MERIT initiative while incorporating valuable lessons learned [9].

The GLEAN initiative is not only a global forum for information sharing and collaboration, but also serves as an action-oriented platform, to reduce the impact that leptospirosis outbreaks have on communities through providing cost-effective, implementable, and sustainable solutions. Within the first years of inception, GLEAN experts made a state-of-the-art assessment of knowledge gaps and developed a strategic framework for future project development [10]. Preliminary outbreak response and control protocols were developed [11]. Additionally, a publication detailing rapid diagnostic tests (RDTs) to detect leptospirosis was written and published with the aim of providing insight into the gaps surrounding RDTs in the field and providing key parameters such as sensitivity and specificity of available diagnostic tests [12]. 

The GLEAN organization structure consists of a steering committee, a secretariat and four working groups comprised of network members to accomplish its goals. The steering committee provides the strategic direction for the secretariat and the member activities. The secretariat is the administrative arm of the organization and provides coordination, financial and human resource management, and performs daily administrative duties. The steering committee is comprised of seven members from international organizations, research laboratories and academic institutions. This includes one member from each of the four working groups. The four working groups are broken down into key technical areas: predict, prevent, detect and intervene. In an effort to promote long-term sustainability of the initiative, emphasis is placed on partnerships who focus primarily on capacity building, operational research, and field operations, all of which are essential to improving community and country level responses to leptospirosis outbreaks.

A cornerstone of the initiative is maintaining accountability among working groups and the steering committee. By ensuring that all members are aware of GLEAN’s mission and the strategic objectives, everyone is able to effectively contribute to the activities to drive a successful initiative. Further, each member, whether they are from non-governmental organizations, foundations, researchers, universities, governments, or international organizations, must have a concrete role within the initiative. This not only ensures all members are engaged, but also guarantees the initiative remains focused on producing results.

A five-year timeline was developed with specific objectives to be accomplished by each of the four working groups. The first year of the initiative was dedicated to accomplishing short-term projects such as conducting a systematic review to identify the location and impact of leptospirosis outbreaks. The focus of subsequent years is on a plethora of issues such as improving the understanding of the relationship between leptospirosis and various associated factors (environmental, biological, ecological, economic and demographic) with a view to providing more timely warnings of the onset of leptospirosis outbreaks and improving the effectiveness of leptospirosis prevention and control strategies.

Each year a technical meeting is held to bring together experts in order to increase global collaboration, share research findings, and translate those findings into operational programs and strategies. The content of the technical meetings is determined by the steering committee in light of the research from the working groups while also taking into account any new research and/or changes in the field. The first GLEAN meeting was held in 2011 in Marseille, France, followed by subsequent meetings in 2012 in Ispra, Italy, and in 2013 in Brasilia, Brazil [10]. 

## 3. Plan of Action

The GLEAN initiative has been organized around four strategic pillars which represent the four primary areas for action as identified by GLEAN members: Predict, Prevent, Detect, and Intervene. The pillars were developed to provide a holistic, multidisciplinary approach with the aim of reducing the overall impact leptospirosis outbreaks have on communities from early detection to timely intervention. GLEAN members are working together to address the issues and answer the critical questions found both within and between the four pillars.

### 3.1. Predict

Predicting when and where outbreaks will occur will be invaluable in preventing the large-scale human health, animal health and socio-economic issues associated with leptospirosis. Prediction and early warning of outbreaks involves an in-depth understanding of the various sectors and factors including climate and weather, environment, vulnerability of populations, rodents and small mammal populations, domestic animals and the water and sanitation systems [11]. 

The five year work plan includes objectives such as determining the baseline disease incidences in pilot sites, determining and evaluating the main determinants of outbreaks and assessing their predictive potential, and developing and testing an instrument for improved outbreak prediction at pilot sites. Currently, operational research is occurring in New Caledonia to understand the interplay between el Niño and leptospirosis outbreaks [13]. Further, exploratory studies are taking place in Morocco in the slaughterhouses in order to understand the relationship between human and animal transmission [14].

### 3.2. Prevent

Preventing leptospirosis outbreaks involves a concerted effort between multiple sectors. Vaccination among domestic animals and human populations needs to be further explored in terms of efficacy and cost-effectiveness. Further investigation into the impact of domestic animal vaccination on human incidence is required. Communication, health education and social mobilization play critical roles in educating at-risk populations on risk factors and the associated preventive actions. Additionally raising awareness of the disease among health care providers is critical. A comprehensive analysis that assesses the effectiveness of human and animal interventions (such as hygiene measures and barrier protection) during an outbreak is necessary. Improving country level capacities, including rodent control and water distribution and sanitation systems, is paramount in preventing the transmission of the disease and in developing cost-effective and sustainable solutions. Additionally, it is important to understand when and how to use weather alerts and forecasts to mitigate the impact of outbreaks [11].

The five year work plan includes objectives such as addressing the efficacy of preventive measures related to human and animal behaviors and environmental source reduction. A long-term goal is to further explore the role of animal and human vaccination. Recently, operational research was conducted in the urban slums of Brazil to understand the impact of repeated exposures to leptospirosis on the communities [15].

### 3.3. Detect

The early and rapid detection of leptospirosis is a critical step in reducing morbidity and mortality. The need to improve clinical confirmation of the disease is important as symptoms are non-specific and the differential diagnosis is wide. Without accurate and available rapid confirmation tests both at the individual and population levels, and appropriate surveillance and alert systems, the detection of outbreaks and seasonal increases as well as distinguishing outbreaks from endemic events becomes difficult. Detecting leptospirosis in rodent, small mammal and domestic animal populations and in the water supply and translating the knowledge into action in order to minimize the impact on human populations requires further evaluation [11]. These investigations are required at a local level, given the wide range of potential serovars, and complex epidemiological factors.

The five year work plan includes designing integrated surveillance systems (including human, animal reservoirs, and water/soil) which will detect outbreaks and will detect changes in carriage/circulation/incidence, developing an improved outbreak detection algorithm in high incidence countries, establishing epidemic threshold levels and case definitions, defining specimen collection and laboratory confirmation procedures, clarifying what type(s) of RDT will be most effective and what the role of laboratory confirmation will be, developing a globally representative serum bank for test comparisons associated with future test evaluations, and developing a lab confirmation protocol. Operational research is being conducted in Tanzania to understand the prevalence of febrile syndromes [16], in Chile to understand prevalence of leptospirosis among rodents in both rural and urban environments [17], and in New Caledonia to understand the prevalence of disease among animals [18]. 

### 3.4. Intervene

Understanding how to effectively respond to leptospirosis outbreaks requires operational research activities. A standardized outbreak investigation and data collection protocol needs to be developed in order to ensure appropriate information is collected during an outbreak. In this way a comprehensive review and analysis can be conducted and evidence based guidance can be provided. This is of importance as outbreaks are often linked to crisis situations which are not favorable for traditional scientific investigations. There are also many questions that remain unanswered both at the community level in terms of controlling the outbreak and at the patient level related to treatment and outcomes. These include how to properly manage cases, when and/or if mass chemoprophylaxis should be administered, and how rodent, small mammal, and domestic animals control should be carried out [11]. 

A five year work plan includes standardizing triage and case management protocols during outbreaks (including addressing severe forms of the disease), improving current outbreak preparedness strategies (including logistics and inter-sectorial plans), and improving the knowledge base related to the impact of public health control measures (chemoprophylaxis, empiric treatment, risk communication, reservoir control, source reduction, barrier intervention, water control, among others). Research is currently being initiated in France to determine the efficacy of chemoprophylaxis on animal models to address the question of distributing chemoprophylaxis to the general population during outbreaks [19]. 

## 4. Institutional Challenges

The GLEAN initiative recognizes the need to develop country and/or regional approaches in order to control leptospirosis. As each country and region has unique challenges, experiences, resources, and response capacities, this tailored approach aims to increase the effectiveness and efficiency of the programs as well as promoting long-term sustainability of the interventions. While the global burden of leptospirosis is far reaching, many regions are still under-represented in the GLEAN initiative. An aim of the steering committee is to increase the involvement of persons, organizations, ministries, and governments in all countries severely impacted by leptospirosis. One aspect of this strategy is to partner with Ministries of Health from countries with a high burden of leptospirosis. In 2013, the Brazilian Ministry of Health hosted the GLEAN meeting which provided an invaluable exchange between researchers and public health professionals across both the American region and globally. Many cross-sectoral collaborations were formed and operational research strategies and implementation techniques were shared. This meeting not only allowed Brazil to showcase its innovative work in the field, such as the knowledge, attitudes, and practices related to Leptospirosis among urban slum residents in Brazil [20], but also allowed international experts to share their experiences. Further, key collaborations were formed as a result of the meeting including a project on rodent control in Brazil. 

Multiple field sites are necessary in order to translate scientific research into operational projects. Currently GLEAN is working to develop partnerships with local governments, institutions and researchers in order to implement and evaluate the multi-disciplinary operational research projects. To date, field sites span a variety of regions, including South America, North Africa and the Pacific. By using field sites in geographically diverse locations the lessons learnt between the various ecosystems, epidemiological settings (urban slums, rural farming areas, nomadic populations with livestock, tropical islands, *etc.*), health systems, and economic/political systems can be shared and adapted in order to save valuable resources. In addition, having specific sites which are solely dedicated to operationalizing the one-health approach allows a variety of disciplines to work together to solve complex research questions and to promote localization and/or nationalization of strategies to suit the various ecological and geographical conditions. 

Like the Merit Initiative [9], funding the GLEAN initiative has proven to be difficult. While there is funding available for pilot projects in the field and bench and operational research, there is minimal funding for secretariat activities. The secretariat is a critical factor in the success of the initiative. It manages day to day activities and finances. This is of utmost importance when multiple sectors and partners around the world are working together because it ensures that no duplication occurs between the projects, and that gaps and opportunities at the community and global level are identified. In the near future, a key function of the secretariat will be to liaise with countries to involve them in GLEAN initiative and to provide them with technical assistance, as required. 

Engagement of the private sector is an additional hurdle. The GLEAN initiative believes engaging partners from project onset to completion and roll-out is critical in order to ensure products are being developed with a public health vision that can be both scalable and cost-effective. For instance, estimates suggest that over 10 million rapid diagnostic tests are needed per annum to meet the current demand for diagnosing suspected cases of leptospirosis. Obtaining buy-in from rapid diagnostic test developers, investors for vaccine development, as well as other innovative solutions and technologies is a slow process and requires an increased awareness of the disease as well as an understanding of the future market potential. 

Since the initiative was developed, GLEAN policies have been continually evolving as knowledge of the disease is gained and additional challenges are identified. When the GLEAN initiative was first established, the priority was on human outbreaks. However, it has become evident that that the level of disease endemicity as well as animal infections and the circulating serovars play a very important role in the explosiveness of human outbreaks; and that by addressing only human outbreaks, a large percentage of the disease burden and underlying problems are being ignored. Addressing the multi-faceted disease with limited resources requires prioritization from the GLEAN initiative as well as involvement and leadership from large international and intergovernmental organizations. 

As leptospirosis is an environmental, occupational, and zoonotic disease, developing large-scale prevention efforts through behavior change and barrier protection remains a challenge. The respective roles of animal and human vaccinations to prevent the disease in the human population needs to still be clarified. A one-health approach has been recognized as the way forward to develop vaccination strategies. In some circumstances, animal vaccination could be considered the most cost-effective [21,22]. However, much more needs to be understood about the dynamics of the disease and the spatial and temporal distribution of the serogroups before a vaccination strategy is defined. 

## 5. Conclusions

Leptospirosis is a globally distributed and highly transmissible disease which is both difficult to diagnose and to treat. Furthermore the conditions which cause the disease to propagate and in which it thrives are poorly understood. The mission of GLEAN is to increase the understanding of the disease and to provide strategic and technological solutions to prevent further spread. The objective is to ensure that the solutions are both cost-effective and sustainable, and that they take into consideration the cross-cultural aspect of the disease. Another important objective of GLEAN is to strengthen the commitment of those already involved and encourage the participation of regions not yet sufficiently represented. This will be achieved through the involvement of governmental organisations, research institutions, and the private sector. In the coming years, the initiative will be challenged with multiple competing priorities and limited funds. However, prioritizing leptospirosis as a public health problem is critical and through increased awareness and support, GLEAN will continue to make strides to fulfil its mission.
